# Clinical efficacy enhancement of a Chinese herbal injection in the treatment of mycoplasma pneumonia in children: A protocol of randomized controlled trial

**DOI:** 10.1097/MD.0000000000025135

**Published:** 2021-03-26

**Authors:** Mian Wang, Hongying Li, Jialing Yang, Meng Wang, Jie Liu

**Affiliations:** a The First People's Hospital of Tianmen City, Tianmen; b The Central Hospital of Wuhan, Wuhan, Hubei Province; c Chongqing Shuangqiao Economic and Technological Development Zone People's Hospital, Chongqing, China.

**Keywords:** azithromycin, combination therapy, mycoplasma pneumonia in children, protocol, randomized controlled trial, tanreqing injection

## Abstract

**Background:**

Mycoplasma pneumonia is a common disease in pediatrics, and macrolides is the first choice for the treatment. However, the increase of antibiotic resistance of macrolides makes it more and more complicated for clinical treatment. Due to the long term treatment of macrolides, it may increase the incidence of nausea, vomiting, abdominal pain, diarrhea, and other gastrointestinal symptoms, vascular phlebitis, liver and kidney function damage. Tanreqing injection, a Chinese herbal extraction injection, has advantages in the treatment of mycoplasma pneumonia in children, and it could improve the curative effect, shortening the course of disease, and reducing the side effects. Yet there is a lack of standard clinical studies to verify it, so this randomized controlled trial (RCT) will evaluate the efficacy and safety of Tanreqing injection combined with azithromycin in the treatment of mycoplasma pneumonia in children.

**Methods:**

This is a prospective RCT to study the efficacy and safety of Tanreqing injection combined with azithromycin in the treatment of mycoplasma pneumonia in children. It is approved by the Clinical Research Society of our hospital. According to the 1:1 ratio, the patients will be randomly divided into Tanreqing injection combined with azithromycin group (observation group) and azithromycin group (control group). Duration of hospitalization, clinical improvement 7 days after admission, changing laboratory tests, pulmonary function, immunoglobulin level, and adverse reactions will be compared between the 2 groups. The data will be analyzed by SPSS 16.0 software.

**Discussion:**

This study will evaluate the efficacy and safety of Tanreqing injection combined with azithromycin in the treatment of mycoplasma pneumonia in children. The results of this experiment will provide clinical basis for the treatment of mycoplasma pneumonia in children with Tanreqing injection combined with azithromycin.

**Trial registration:**

OSF Registration number: DOI 10.17605/OSF.IO/X6VFS.

## 1 Introduction

Mycoplasma pneumonia (MP) is one of the common respiratory diseases in clinic, which mainly occurs in children and adolescents. The main clinical manifestations are fever, cough, and lung rale.^[1]^ In recent years, the number of pediatric patients infected with Mycoplasma pneumoniae is increasing year by year, and showing a younger trend.^[2]^ According to the survey, MP accounted for 10% to 40% of hospitalized children with community-acquired pneumonia.^[3]^ Because of the serious side effects of tetracycline and fluoroquinolones on children, macrolides have become the first choice for the treatment of mycoplasma pneumonia in children.^[4,5]^ Azithromycin (a macrolide antibiotic) is the first choice, which has a long half-life and obvious effect on target cells.^[6]^ It has the advantages of strong inhibition to mycoplasma, little damage to liver and kidney function, and little stimulation to stomach. However, the increase of antibiotic resistance of macrolides makes it more and more complicated for clinical treatment,^[7,8]^ also, the long term treatment of azithromycin may increase the incidence of nausea, vomiting, abdominal pain, diarrhea and other gastrointestinal symptoms, vascular phlebitis, so it is necessary to seek new alternative treatments.

Traditional Chinese medicine injection is made of ingredients extracted from Chinese herbal medicine by modern technology, which has the characteristics of rapid action and high bioavailability.^[9]^ It has been confirmed that traditional Chinese medicine injection combined with azithromycin in the treatment can improve the efficacy and safety of MP in children.^[10]^ Tanreqing is a traditional Chinese medicine injection extracted from Scutellaria baicalensis, bear bile powder, goat horn, honeysuckle, and forsythia suspense. It has the effect of reducing fever, resolving phlegm, and relieving cough. It can not only inhibit a variety of viruses and bacteria, but also regulate immune function and enhance immunity.^[11]^ Its efficacy and safety have been confirmed in influenza, lung infection, and other diseases.^[11,12]^ Tanreqing injection has been used in the treatment of mycoplasma pneumonia in children for many years, and it could improve the curative effect, shortening the course of disease, and reducing the side effects of Azithromycin.^[13]^ However, there is no strict randomized controlled trial (RCT) to study the efficacy of Tanreqing injection combined with azithromycin in the treatment of MP in children. Therefore, we intend to use this RCT to evaluate the efficacy and safety of Tanreqing injection combined with azithromycin in the treatment of mycoplasma pneumonia in children.

## 2 Materials and methods

### 2.1 Study design

**Figure 1:**
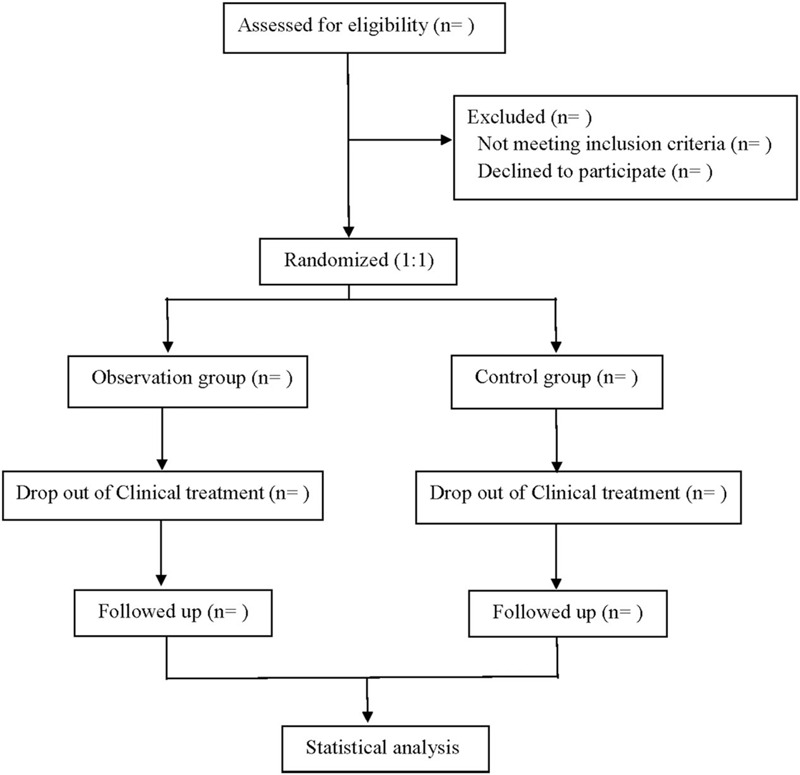
Flow diagram.

This is a single center, prospective RCT to study the efficacy and safety of Tanreqing injection combined with azithromycin in the treatment of mycoplasma pneumonia in children. The study will be based on the SPIRIT Randomized study list, follow the Consolidated Standards of Reporting Trials, and provide Consolidated Standards of Reporting Trials flow charts (Fig. 1).

### 2.2 Ethics and registration

This research scheme is in line with the Helsinki Declaration and approved by the Clinical Research Ethics Committee of our hospital. This experiment has been registered in the open science framework (OSF) (registration number: DOI 10.17605/OSF.IO/X6VFS). Before the beginning of the study, we will fully inform the family members of the patients about the purpose, method and potential risks of this study, and sign a written informed consent form after obtaining their consent. They are free to choose whether to continue the experiment at any time during the study.

### 2.3 Patients

Inclusion criteria:

1. meet the diagnostic criteria of mycoplasma pneumoniae pneumonia in children, age ≤15 years old;
2. have obvious clinical symptoms of irritant cough, and chest imaging examination to support the diagnosis;
3. serum mycoplasma pneumoniae specific antibody IgM test showed positive.

Exclusion criteria:

1. complicated with other respiratory tract infection;
2. liver and kidney function damage;
3. allergy to azithromycin or Tanreqing injection;
4. unable to cooperate with health care workers to carry out relevant diagnosis and treatment in the course of treatment;
5. patients with test drug allergy;
6. patients with severe digestive or blood system abnormalities;
7. recent treatment with immunosuppressants and other antibiotics;
8. unable to understand the research scheme or unwilling to participate after explanation.

### 2.4 Distribution scheme

We plan to include 120 patients who meet the requirements, which are randomly divided into the observation group and the control group according to the ratio of 1:1. Random numbers will be generated by computer software programs designed by statisticians who are not familiar with the content of the study. The random numbers assigned to each group will be written on a piece of paper and placed in a sealed and opaque envelope. Envelopes will be provided to participants in random order. The evaluators in the trial do not know the random assignment of the participants. The statistics and analysis of the data will be completed by statistical researchers who have no knowledge of the grouping.

### 2.5 Intervention measures

After admission, all patients will receive symptomatic and support treatment, such as antipyretic, antitussive, expectorant, oxygen inhalation, and so on. The following treatment plan according to the group will be performed.

Control group: Azithromycin sequential therapy, give azithromycin injection, 10 mg/ (kg d) + 5% glucose injection, 100 ml to 250 ml intravenous drip, once a day. After continuous use for 3 days, stop for 4 days, and then give azithromycin dry suspension 10 mg/kg, orally, once a day for 7 days.

Observation group: on the basis of the treatment plan of the control group, Tanreqing injection will be given 0.5 ml/ (kg d) + 5% glucose injection 100 ml intravenous drip, once a day, and the daily threshold is 20 ml, for 14 days.

The distribution of drugs will be distributed by unwitting nurses to patients with corresponding numbers according to the drug number. The patients will be treated according to the corresponding treatment plan for 2 wk and followed up for 30 d. Lung function, laboratory examination, and chest imaging examination will be performed 1 week after treatment and the end of treatment.

### 2.6 Observation index

1. The primary outcomes are duration of hospitalization, and clinical improvement 7 days after admission.
2. The secondary outcomes are changing laboratory tests (IgA, IgG, and IgM) during 7 days, lung function, changing of CT findings after 30 days.
3. Adverse reactions include gastrointestinal reactions, rash, dizziness, abnormal liver and kidney function, etc.

### 2.7 Data collection and management

The whole process of data collection and recording will be recorded by 1 or 2 assistants. Personal information about potential participants and registered participants will be collected, shared and stored in a separate storeroom to protect confidentiality before, during and after the trial. Only the researchers of this research group have access to the relevant data of the study.

### 2.8 Statistical analysis

The data will be analyzed by SPSS 16.0 software. The data of continuous variables of normal distribution will be tested by independent design *t* test, the data of continuous variables of skewed distribution will be tested by Mann–Whitney *U* test, and the data of classified variables will be tested by Chi-Squared test, and the difference is statistically significant when *P* *<* *.05.*

## 3 Discussion

MP is an inflammation of alveoli, airways, or pulmonary interstitium caused by mycoplasma infection. It is a common pediatric disease,^[14,15]^ which can show a concentrated outbreak. The disease has the characteristics of urgent onset, rapid progress, critical condition, and easy recurrence, which seriously endangers the health of children.^[16]^

Azithromycin is a macrolide antimicrobial agent, and mycoplasma is more sensitive to it. But long-term intravenous injection may bring more adverse reactions, especially in children. Although sequential therapy can reduce adverse reactions, the possible drug resistance directly affects its efficacy.^[17]^ Tanreqing injection is a preparation of traditional Chinese medicine. Modern pharmacological studies have reported that Tanreqing injection can reduce the levels of tumor necrosis factor, interleukin (IL)-1, IL-6, and IL-8, inhibit the expression of inflammatory cytokines, improve airway mucus hypersecretion and airway inflammatory injury, and have anti-inflammatory, antibacterial and antiviral effects.^[18]^ The combination of the 2 drugs will improve the therapeutic effect, and studies have confirmed that the treatment scheme of integrated traditional Chinese and Western medicine is economical, safe, and effective.^[19]^ This study will evaluate the efficacy and safety of Tanreqing injection combined with azithromycin in the treatment of mycoplasma pneumonia in children through a strict randomized controlled scheme, so as to provide clinical basis for the promotion of this scheme.

As this study is a single center study, the small sample size and the regionalization of the included population may have a certain impact on the results, so more RCTs with more centers and large samples are needed to verify our conclusions.

## Author contributions

**Data collection:** Hongying Li and Meng Wang

**Funding support:** Jialing Yang

**Investigation:** Meng Wang

**Resources:** Meng Wang and Jie Liu

**Software operating**: Jie Liu

**Supervision:** Hongying Li and Jialing Yang

**Writing – original draft:** Mian Wang and Hongying Li

**Writing – review & editing:** Mian Wang and Jialing Yang
